# Limited evidence to recommend against open chest cardiopulmonary resuscitation in blunt trauma

**DOI:** 10.1186/s13054-017-1831-x

**Published:** 2017-09-20

**Authors:** Stefano Malinverni, Pierre Mols

**Affiliations:** Emergency Department, Centre Hospitalier Universitaire Saint Pierre, Université Libre de Bruxelles, Rue Haute 322, 1000 Brussels, Belgium

## Main Text

We congratulate Endo and co-authors on their retrospective registry-based analysis comparing closed-chest cardiopulmonary resuscitation (CCCPR) and open-chest cardiopulmonary resuscitation (OCCPR) in cardiac arrest patients following blunt trauma [1] and would like to share the following comments.

OCCPR is generally deemed futile in blunt trauma patients whenever cardiopulmonary resuscitation (CPR) is ongoing for more than 10 minutes without the patient showing any signs of life [2]. Among patients for whom OCCPR is recommended expected survival doubles whenever the patient is in profound shock compared to patients without vital signs [2]. The lack of information about CPR duration and circulatory status before OCCPR initiation jeopardizes the interpretation of the overall results as great heterogeneity might exist between the two propensity-score matched groups. It is potentially misleading to draw conclusions about efficacy of a treatment (OCCPR) whenever indications of the aforementioned treatment cannot be assessed.

A further confounding element is that OCCPR is initiated in most of the cases for logistic reasons after initial treatment by CCCPR. During this initial window period a substantial proportion of potentially salvageable patients might achieve return over spontaneous circulation (ROSC) [3]. Early ROSC is associated with increased survival [4], and it precludes any further treatment by OCCPR. Therefore, patients from this registry treated through OCCPR should be considered as having an intrinsically worse prognosis at treatment initiation despite rigorous stratification and matching.

The results would be more consistent if duration of CCCPR before OCCPR were known. This would allow exclusion from the analysis of cases where ROSC occurred before the average duration of CCCPR at OCCPR initiation.

While acknowledging that the utilization of an instrumental variable (IV) might be used to control for unmeasurable confounders in non-randomized experiments, its choice should nevertheless respect the fundamental assumption that the IV is not directly associated with the outcome variable [5]. The IV “mean number of OCCPR cases per year in the hospital” captures hospital characteristics in a given year, which can be directly linked to survival as high-volume centers in terms of OCCPR are probably associated with increased resuscitation skills through this technique. This invalidates the use of the IV to measure OCCPR implementation.

In our opinion OCCPR in blunt trauma cannot be rigorously associated with reduced rates of hospital discharge and of 24-h survival following Emergency Department admission based on the available evidence.

## Abbreviations

CCCPR: Closed-chest cardiopulmonary resuscitation
CPR: Cardiopulmonary resuscitation
EDT: Emergency department thoracotomy
IV: Instrumental variable
OCCPR: Open-chest cardiopulmonary resuscitation
ROSC: Return over spontaneous circulation

## References

[1] Endo A, Shiraishi A, Otomo Y, Tomita M, Matsui H, Murata K (2017). Open-chest versus closed-chest cardiopulmonary resuscitation in blunt trauma: analysis of a nationwide trauma registry. Crit Care.

[2] Burlew CC, Moore EE, Moore FA, Coimbra R, McIntyre RC, Davis JW (2012). Western Trauma Association critical decisions in trauma: resuscitative thoracotomy. J Trauma Acute Care Surg..

[3] Goto Y, Funada A, Goto Y (2016). Relationship between the duration of cardiopulmonary resuscitation and favorable neurological outcomes after out‐of‐hospital cardiac arrest: a prospective, nationwide, population‐based cohort study. J Am Heart Assoc.

[4] Rajan S, Folke F, Kragholm K, Hansen CM, Granger CB, Hansen SM (2016). Prolonged cardiopulmonary resuscitation and outcomes after out-of-hospital cardiac arrest. Resuscitation..

[5] Rassen JA, Schneeweiss S, Glynn RJ, Mittleman MA, Brookhart MA (2009). Instrumental variable analysis for estimation of treatment effects with dichotomous outcomes. Am J Epidemiol..

[6] Kozower BD, Stukenborg GJ (2017). Volume-Outcome Relationships in Thoracic Surgery. Thorac Surg Clin..

[7] Hamidian Jahromi A, Northcutt A, Youssef AM (2013). A Patient With Blunt Trauma and Cardiac Arrest Arriving Pulseless at the Emergency Department; is that Enough Reason to Stop Resuscitation? Review of Literature and Case Report. Iran Red Crescent Med J..

