# Supplementary material for: Limited evidence to recommend against open chest cardiopulmonary resuscitation in blunt trauma
Source: Crit Care. 2017 Sep 20;21:244. doi: 10.1186/s13054-017-1831-x (PMC5607573; doi:10.1186/s13054-017-1831-x)
Supplement: Related texts

# Reply: Authors’ response

**Authors:** Akira Endo, Atsushi Shiraishi, Yasuhiro Otomo

We would like to thank Malinverni et al. for their comments regarding our recent article [1]. We had to compromise on the study design as a retrospective observational study since this research question could not be evaluated by a randomized controlled trial due to ethical issues. Although we adjusted for the biases as much as possible using the best conceivable statistical models for causal inferences, several unsolvable problems remained due to the study design.

Information on the duration of CCCPR before the implementation of OCCPR was lacking in the Japan Trauma Data Bank, and could be considered as an unmeasured confounder. However, we performed subgroup analysis according to the timings of cardiac arrest (at the scene of injury and upon arrival at the emergency department) and pre-hospital transport time, which were considered to be greatly associated with CCCPR duration before OCCPR, and could be surrogate variables. Interestingly, these subgroup analyses did not show significant interactions and did not have any trends for favorable/unfavorable outcomes, indirectly suggesting that the impact of CCCPR duration before OCCPR on in-hospital survival was limited. As for the concern regarding the IV we used, to our knowledge, the volume–outcome relationships regarding OCCPR have not been reported previously, and these relationships do not always exist in surgical procedures [6]. It is considered to be insufficient to deny eligibility of the IV based on unfounded assumptions. At least, it can be said that our research failed to show the effectiveness of OCCPR in multiple models adjusted as much as possible for measured/unmeasured biases.

We would like to emphasize that we did not evaluate the effectiveness of emergency department thoracotomy (EDT), but only a procedure of OCCPR that is a part of EDT, which includes hemostasis of intra-thoracic organs and/or aortic cross-clumping. EDT is occasionally also performed in patients without cardiac arrest. Several reports have described cases where patient survival was dependent on EDT including OCCPR [7]; we have also experienced such cases.

However, we should recognize that the evidence supporting the effectiveness of OCCPR remains scarce, at least among blunt trauma patients as a whole, and we have to conduct further studies to assess a limited specific population who benefit from EDT including OCCPR.
